# Surface Active Agents and Their Health-Promoting Properties: Molecules of Multifunctional Significance

**DOI:** 10.3390/pharmaceutics12070688

**Published:** 2020-07-21

**Authors:** Ioannis Anestopoulos, Despoina Eugenia Kiousi, Ariel Klavaris, Alex Galanis, Karina Salek, Stephen R. Euston, Aglaia Pappa, Mihalis I. Panayiotidis

**Affiliations:** 1 Department of Molecular Biology & Genetics, Democritus University of Thrace, 68100 Alexandroupolis, Greece; ianestop@mbg.duth.gr (I.A.); dkiousi@mbg.duth.com (D.E.K.); agalanis@mbg.duth.gr (A.G.); 2 Department of Biological Sciences, University of Cyprus, 2109 Nicosia, Cyprus; klavaris.ariel@ucy.ac.cy; 3 Institute of Mechanical, Process & Energy Engineering, Heriot Watt University, Edinburgh EH14 4AS, UK; k.salek@hw.ac.uk (K.S.); s.r.euston@hw.ac.uk (S.R.E.); 4 Department of Applied Sciences, Northumbria University, Newcastle Upon Tyne NE1 8ST, UK; 5 Department of Electron Microscopy & Molecular Pathology, The Cyprus Institute of Neurology & Genetics, 2371 Nicosia, Cyprus; 6 The Cyprus School of Molecular Medicine, P.O. Box 23462, 1683 Nicosia, Cyprus

**Keywords:** surface active agents, surfactants, health promotion, anti-microbial, anti-oxidant, anti-viral, anti-inflammatory, anti-cancer, anti-aging

## Abstract

Surface active agents (SAAs) are molecules with the capacity to adsorb to solid surfaces and/or fluid interfaces, a property that allows them to act as multifunctional ingredients (e.g., wetting and dispersion agents, emulsifiers, foaming and anti-foaming agents, lubricants, etc.) in a widerange of the consumer products of various industrial sectors (e.g., pharmaceuticals, cosmetics, personal care, detergents, food, etc.). Given their widespread utilization, there is a continuously growing interest to explore their role in consumer products (relevant to promoting human health) and how such information can be utilized in order to synthesize better chemical derivatives. In this review article, weaimed to provide updated information on synthetic and biological (biosurfactants) SAAs and their health-promoting properties (e.g., anti-microbial, anti-oxidant, anti-viral, anti-inflammatory, anti-cancer and anti-aging) in an attempt to better define some of the underlying mechanism(s) by which they exert such properties.

## 1. Introduction

Surfactants are amphiphilic molecules characterized by a hydrophilic (ionic or non-ionic) head group and a hydrophobic tail. Biosurfactants are simply surfactants produced by living organisms. Surfactants are classified based on their head group polarity into non-ionic (uncharged), anionic (negatively charged), cationic (positively charged) and zwitterionic (capable of possessing both positive and negative charge). This complexity of head group leads to a range of adsorption and solution behaviors that facilitates their use across a broad range of industrial sectors. The classic application of surfactants is as surface-active agents (SAAs) which exploits their ability to adsorb to solid surfaces and fluid interfaces by partitioning the hydrophilic part in the aqueous phase and the hydrophobic part in/or at the second phase. This allows SAAs to act as wetting and dispersion agents, emulsifiers, foaming and anti-foaming agents and lubricants in a wide range of consumer and other industrial products such as detergents and soaps, foods, pharmaceuticals, cosmetics and personal care products, herbicides, insecticides and sanitizers [1]. It has also been realized that their rich and diverse chemistry imparts a range of bioactive properties including anti-microbial, anti-oxidant, anti-viral, anti-inflammatory, anti-cancer and anti-aging. This is important for surfactant manufacturers as it extends the market for SAAs and allows them to be branded as multi-functional ingredients. Similarly, consumer product manufacturers can make additional bioactive claims against products containing surfactants, which enhance the appeal and benefits to consumers and increases sales and market share. As importantly, multi-functional ingredients can lead to potential cost savings for the manufacturers of consumer formulations. For example, SAAs are used as emulsifiers in many foods, as well as cosmetic and pharmaceutical formulations. Having both the surface active as well as the anti-microbial properties of SAAs being exploited, this would remove (or reduce) the need for additional preservatives to be added to the final consumer product(s), with a concomitant decrease in formulation costs.

The origin of these bioactive properties most likely arises from the ability of SAAs to interact with proteins and lipid membranes. The propensity for SAAs to bind either to specific binding sites or non-specifically to proteins (including enzymes) is wellknown [2], and they have been exploited as protein denaturants, for many years, in sodium dodecyl sulfate–polyacrylamide gel electrophoresis (SDS-PAGE) assays. Thus, there is certainly the possibility that SAAs can and will disrupt critical microbial and cellular metabolic processes in addition to interacting with membranes [3]. The amphiphilic structure of SAAs allows them to penetrate the phospholipid leaflet of a membrane, modifying the membrane structure and fluidity, thus increasing the permeability and disrupting cellular metabolic processes.

Biosurfactants are receiving increasing attention mainly due to the perception that they are natural ingredients, a selling point appealing to both manufacturers of products and consumers. It has also become apparent that biosurfactants have other appealing properties that differentiate them from their synthetic counterparts, such as higher biodegradability and lower toxicity and ecotoxicity, thus further enhancing their green, natural credentials [4].

In this review, the veracity of the scientific evidence for the bioactive properties of a range of biological and synthetic surfactants is considered, with an emphasis on the mechanism(s) of action and their potential use as the components of industrial and consumer product applications. The discussion on biosurfactants will be confined to those that are produced by microorganisms (bacteria, fungi, yeast) and in particular the glycolipids (rhamnolipids, sophorolipids and mannosityl erithritol lipids (MELs)).

## 2. Structure of Synthetic and BioSurfactants

**Figure 1:**
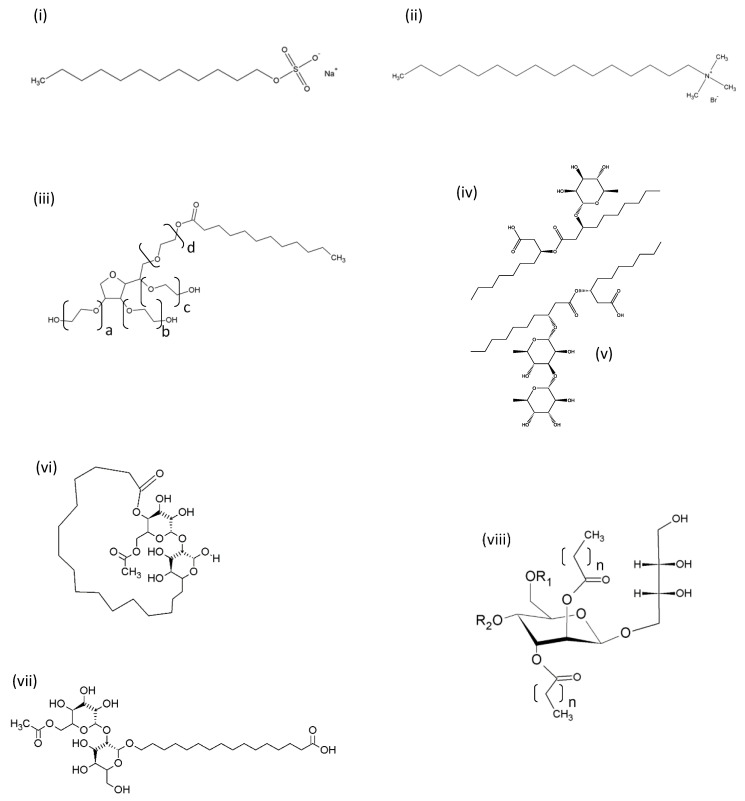
Representative structures of synthetic and biosurfactants. (**i**) Anionic synthetic surface-active agent (SAA) sodium dodecyl sulphate (SDS); (**ii**) cationic synthetic SAA cetyltrimethyl ammonium bromide (CTAB); (**iii**) non-ionic synthetic SAA Tween 20 (polyoxyethylene sorbitan monolaurate) (Tween 40 = monopalmitate; Tween 60 = monostearate; Tween 80 = monooleate, the sum of alkyl chains a, b, c and d is 20 for all Tweens); (**iv**) Monorhamnolipid R1 with two C10 alkanoate chains; (**v**) Dirhamnolipid R2 with two C10 alkanoate chains; (**vi**) Acetylated lactonic sophorolipid; (**vii**) Acetylated acidic sophorolipid; (**viii**) Mannositylerithritol lipid (the length of the alkyl chains, n, can be between 6 and 10). For mannosityl erithritol lipid (MEL), A, R_1_ and R_2_ are acetyl groups; MEL B, R_1_ = acetyl, R_2_ = H; MEL C, R_1_ = H, R_2_ = acetyl.

Synthetic and biosurfactants are amphiphiles, which have both hydrophilic and lipophilic regions that allow them to adsorb to interfaces, including biological membranes. Their structures, however, can differ markedly, particularly in terms of their hydrophilic regions. Synthetic surfactants often contain an alkyl chain attached to a polar or compact charged hydrophilic head group [5]. Glycolipid biosurfactants, on the other hand, have one or two sugar rings as amore bulky polar head group (typically rhamnose, sophorose, galactose, mannosylerithritol, trehalose) [4] which can differentiate their properties from their synthetic counterparts. Figure 1 gives a comparison of typical non-ionic, anionic, cationic, zwitterionic and glycolipid surfactants.

## 3. Anti-Microbial Properties

The discovery and production of anti-microbial agents at the industrial scale has significantly contributed to the world-wide increase in life-expectancy and to the improvement of life quality. Antibacterial and antifungal factors can eliminate the viability and growth of microbial populations by: (i) disrupting their extracellular membranes and/or their cell wall (e.g., penicillin), (ii) inhibiting gene expression (e.g., aminoglucosides), (iii) inflicting DNA damages (e.g., metronidazole) or (iv) manipulating important metabolic pathways (e.g., sulfonamides). However, in recent decades, the severity of antibiotic resistance has dramatically increased, while the discovery of novel safe antibiotics has plummeted. A meta-analysis conducted in 2014 has shown that antibiotic abuse in hospital and community settings is the main factor leading to resistance [6]. Today, the most resistant bacteria associated with severe hospital-acquired infections (HAI) are *Enterococcus faecalis, Staphylococcus aureus, Klebsiella pneumoniae, Acinetobacter baumanii, Pseudomonas aeruginosa* and *Enterobacter* sp. which can often result in fatal outcomes. In addition, some of the aforementioned bacteria as well as fungi (e.g., *Candida albicans*) can organize into compact structures (i.e., biofilms) that attach to abiotic or biotic surfaces. Biofilms are comprised of microorganisms with an altered metabolic activity that are enclosed in a calyx of exopolysaccharides, DNA and proteins. Neither the host’s immune cells nor antibiotics can penetrate these structures, thus leading to chronic infections [7]. Biofilms can cause serious implications in the recipients of prosthetics and patients with chronic wounds, as the bacteria can be dislodged from the structure, travel through the bloodstream and possibly establish bacteremia and systemic infections. Today, antibiotic use, infection control and the development of novel disinfectants and vaccines are the main challenges for the scientific community. To these ends, compounds with surface-active properties are considered promising antibiotic/disinfectant agents, as well as antibiotic-delivery vehicles due to their physicochemical properties. Most of these molecules disrupt the outer and inner membranes of pathogens by exploiting their charge and hydrophobicity. The advantages of using SAAs as anti-microbials include their broad-spectrum bactericidal action and the lack of resistance mechanisms from the pathogens. Nonetheless, a major setback for the medical administration of these agents is their potential hemolytic activity, due to the lack of specificity of action. 

The largest category of synthetic surfactants with anti-microbial actions is cationic surfactants, which exhibit a broad spectrum of biostatic and biocidal activities against planktonic pathogens. They exert their actions by electrostatically binding onto the negatively charged molecules of bacterial surface and disrupting the membrane continuity as well as the metabolic processes that take place on the bacterial inner membrane. However, these surfactants are mainly effective against Gram-positive bacteria and fungi, while most Gram-negative bacteria are resistant. Some examples of such compounds are: (i) a hexameric surfactant with amide moieties shown to be effective against *E. coli* [8], (ii) an amide gemini cationic surfactant 12 (AGS12) with incorporated silver nanoparticles shown to limit the growth of *Bacillus subtilis, Staphylococcus aureus, E. coli, P. aeruginosa, C. albicans* and *Aspergillus niger* [9], (iii) V-16 and VBP-16 (two dicationic surfactants containing viologen, vinylbipyridinium moieties and hexadecyl chains) exhibiting inhibitory properties against *S. aureus* 209P, *B. cereus* 8052 and *C. albicans* 855-653 [10] and (iv) lysine-derived mono-catenary or gemini surfactants with anti-*S. aureus, Staphylococcus epidermidis* and *B. subtilis* activity [11]. Other surfactants and surfactant emulsions rely on their hydrophobic moieties for their anti-microbial activity. These compounds are usually more efficient in combating Gram-negative bacteria than cationic surfactants. For example, benzalkonium chloride (BAC) analogues with hydrophobic chains of varying length exhibited anti-microbial activity against (i) Gram-positive bacteria *S. aureus* and *E. faecalis*, (ii) Gram-negative bacteria *E. coli* and *P. aeruginosa* and (iii) fungus *C. albicans* [12] while other nonionic, micelle-forming silicon polyether surfactants (with short hydrophilic chains that form micelles) were shown to be effective against *E. coli* B21 [13]. Similarly, *N^a^*-lauroyl-arginine methyl ester-derived cationic double-chain surfactants (with arginine heads) rely on their lipophilic moietiesfor their anti-microbial activity. Indeed, the most lipophilic analogue, LANHC_X_, exhibited profound inhibitory activity against *Micrococcus luteus* ATCC 9341, *S. aureus* ATCC 29213 and *S. epidermidis* ATCC 12228 and a weaker one against *P. aeruginosa* ATCC 27853, *C. albicans* ATCC 10321 and *E. coli* ATCC 25922 [14]. Lastly, gemini lipopeptide surfactants (with aliphatic chains and Lys residue(s) at the Y- and/or Z-position) exhibited anti-microbial activity against *E. coli* (K12 and W3110), *Salmonella typhimurium* LT2, *B. subtilis* 168 and *S. aureus* FDA 209P. Importantly, these surfactants can, specifically, target the bacterial membranes and do not cause hemolysis to rabbit red blood cells [15].

With glycolipid biosurfactants, there is evidence that the mechanism of antimicrobial activity depends on the glycolipid type, evidenced by the rhamnolipids inhibiting bacterial growth in the exponential phase whilst sophorolipids inhibit growth between the exponential and stationary phases [16]. Diaz de Renzo et al. [16] suggest that this may be due to the way the two biosurfactants interact with the cell membrane, with rhamnolipids having a greater ability to insert their acyl chains into thus disrupting the membrane [17], whilst the mechanism of action of sophorolipids is closer to that of antibiotic drugs. There is also evidence that rhamnolipids and sophorolipids have differing activity against Gram-positive and Gram-negative bacteria, with any activity being strongly pH dependent. *Salmonella enteric* and *Escherichia coli* (both Gram-negative) are resistant to rhamnolipids at all pH [18]. In a separate study, the Gram-negative opportunistic pathogen Pseudomonas aeruginosa PAO1 was also shown to be unaffected by rhamnolipid [16]. On the other hand, the Gram-positive *Listeria monocytogenes*, *Bacillu cereus* and *Staphylococcus aureus* all show susceptibility to rhamnolipid, with *B. cereus* the most sensitive and *S. aureus* more sensitive at a low pH [16,18,19]. Oral pathogens of the genus Streptococcus (*S. mutans, S. oralis, S. sanguinis*), *Actinomyces naeslundii* and *Neisseria mucosa* are also sensitive to rhamnolipids in both the planktonic and biofilm state [20].

Sophorolipids have been found to have a bactericidal effect towards Gram-positive and Gram-negative strains [21]. Diaz De Rienzo et al. [22] presented an inhibitory effect of sophorolipids (SLs) on the growth of Gram-positive *Bacillus subtilis* BBK006 and Gram-negative *Cupriavidus necator* ATCC 17699, as well as the biofilm-disruption properties on the biofilms of single and mixed cultures of *B. subtilis* BBK006 and *Staphylococcus aureus* ATCC 9144. In the latest study by Ceresa et al. [23], the antimicrobial properties of SL in medical-grade silicone discs were evaluated against *Staphylococcus aureus* ATCC 6538, *Pseudomonas aeruginosa* ATCC 10145 and *Candida albicans* IHEM 2894. SLs were found to significantly decrease a biofilm formation by both Gram-positive strains and significantly reduce the attachment of the yeast *C. albicans* on the silicone-coated discs. Sophorolipids adsorbed onto gold surfaces showed a compelling reduction in the viability of some significant Gram-positive (*Enterococcus faecalis*, *Staphylococcus epidermidis*, *Streptococcus pyogenes*) and Gram-negative (*Escherichia coli*, *Pseudomonas aeruginosa* and *Salmonella typhymurium*) pathogens, as reported by Valotteau et al. [24]. These reports feed into a potential application of SLs in antiadhesion therapy, which is proposed as an alternative to the use of antibiotics and antifungal drugs for treating biofilm-associated infections [25]. According to the authors, SLs exhibited strong and versatile antiadhesion properties against two well known nosocomial pathogens, *Staphylococcus aureus* and *Escherichia coli*, making these surfactants promising candidates for the design and production of anti-infective biomaterials.

The antibacterial activity of MELs against Gram-positive sporing bacterium *Bacillus cereus* was reported by Shu et al. [26]. *B. cereus*, which is a food-borne pathogen, is able to produce spores resistant to high temperatures, UV and chemicals; therefore, the authors targeted both the vegetative cells as well as the spores for treatment with MELs. Their findings show that MELs had a significant effect on the spores’ germination and also successfully reduced the viability of vegetative cells. Their findings show a high potential of MELs as a natural preservative in the food industry. One of the recent studies by Ceresa et al. [27] presented an excellent activity of MELs against a wellknown pathogen *Staphylococcus aureus* ATCC 6538. The surfactants were efficient at disrupting the *S. aureus* biomass, reducing the metabolic activity of the biofilm and also showed bacteriostatic and bactericidal properties against the pathogen. The biofilm experiments were performed on silicone-coated discs. 

Janek et al. [28] examined the antimicrobial and antiadhesive properties of trehalose lipids (TLs) against a number of pathogens. The authors concluded that TLs indicated the highest antimicrobial activity against *Vibrio harveyi* and *Proteus vulgaris*, as well as inhibited the growth of the yeast *Candida albicans* by 30%. Moreover, the authors observed a strong antiadhesive property, especially against *C. albicans* and *Escherichia coli* on polystyrene surfaces and silicone urethral catheters. According to the authors, their findings show that TLs could be successfully used as surface-coating agents.

Surfactants could be effective anti-biofilm agents due to their physicochemical properties that allow them to penetrate and disrupt hydrophobic structures. For instance, (i) sodium hypochlorite (SHY) effectively disrupted *Listeria monocytogenes* biofilms and killed its planktonic cells [29], (ii) cetylpyridinium chloride exerted bactericidal effects against planktonic cells and the biofilms of clinically relevant cariogenic *Streptococcus* species [30], whereas (iii) cetylpyridium chloride nano-emulsion limited the attachment of other cariogenic strains like *Streptococcus mutans*, *Lactobacillus casei*, *Actinomyces viscosus* and *C. albicans* [31]. In the same manner, novel synthesized ionic liquid nanoparticles [(IL)-incorporated 1-butyl-3-methylimidazolium hexafluorophosphate-incorporated, chitosan-modified, submicron-sized poly (DL-lactide-co-glycolide; PLGA)] together with surfactants Tween-20 or Poloxamer-188, managed to eliminate *S. epidermidis* biofilms. The underlined mechanism involved the degradation of the biofilms’ polysaccharides (EPS) content, thereby exposing the underlying bacteria to the action of the surfactants, consequently inducing the destruction of the cell wall and the cytoplasmic membrane as well [32]. Additionally, the synthetic surfactants could act in synergy with other surfactants and/or antibiotics in order to maximize their beneficial outcomes. To this end, a 8:1 mixture of arginine-based surfactants [NαNω-bis (Nαcaproylarginine)] α, ω-propyldiamide [C_3_(CA)_2_]] together with an anionic lipopeptide (lichenysin), synergistically limited the growth of entero-pathogens *Yersinia enterocolotica*, *E. coli* O157:H7, *B. subtilis* and *C. albicans*, due to their ability to interact with bacterial phospholipids and to activate signaling cascades resulting in a viable, but not culturable, state [33]. Moreover, the gemini quarter ammonium bromides [10 (TMPAL-10 Br) and 12 (TMBEAL-12 Br)] were shown to act synergistically with azoles (itraconazole and fluconazole) or polyenes (nystatin and amphotericin), two classes of antifungal agents, resulting in a two-fold increase in the surfactant’s inhibitory activity against the pathogens *C. albicans* and *S. cerevisiae*. In addition, both TMPAL-10 Br and TMBEAL-12 Br exerted an antiadhesive action, while only TMEAL-12 Br could dislodge pre-formed *C. albicans* biofilms in a dose-dependent manner. Finally, TMPAL-10 completely stopped filament formation in *C. albicans*, a key early process for biofilm formation [34].

Biosurfactants are also the potent disruptors of biofilms for human bacterial and yeast pathogens. Rhamnolipids were able to remove up to 88.9% of the *S. aureus* biofilm in skim milk or 35% in nutrient broth, both deposited onto polystyrene [35]. Similarly, Aleksic et al. extracted dirhamnolipids (with two C10 acyl chains, C10C12 and C8C10 acyl chains) from Lysinibacillus sp. BV152.1 fermentations, and showed that they could prevent biofilm formation by *P. aeruginosa* PAO1, *S. aureus*, and *Serratia marcescens* [36]. The semi-synthetic morpholine amide derivative of these rhamnolipids showed an increased biofilm disruption activity (80% against 50% for the unreacted rhamnolipids) with *P. aeruginosa* PAO1. Rhamnolipid congener type also affects biofilm disruption with C10C10 mono-rhamnolipids produced by *P. aeruginosa* having a lower effect than C14C14 di-rhamnolipids from *Burkholderia thailandensis* E264 on biofilm formation by *Bacillus subtilis* BBK006 [37]. Bisurfactants also appear to be less effective at preventing or removing biofilm in mixed bacterial cultures compared to single strains. Rhamnolipids at 0.25% were able to remove 24% of the biofilm from a mixed culture of *S. aureus*, *L. monocytogenes* and *S.Enteritidis*, compared to 58.5% for *S. aureus*, 26.5% for *L. monocytogenes*, and 23.0% for *S.Enteritidis* on their own [38]. The biofilm-disrupting ability of rhamnolipids extends to pathogenic fungi. Rhamnolipids at 0.16mg/L reduced the adhesion of *C. albicans* to polystyrene by 50%, increasing to 90% at 5mg/L [39]. With *Yarrowia lipolytica*, rhamnolipid is more efficient than CTAB or SDS at reducing biofilm formation (up to 50%) [40].

Importantly, surfactants and emulsions can be incorporated into chronic wound care either as direct anti-microbial agents, or due to their ability to produce gels and foams for topical application. Chronic wounds usually attract biofilm-forming microorganisms that decelerate wound healing and often lead to systemic disorders. In this context, a non-propellant based foam (NPF; loaded with the antibiotics sulfadiazine and pectin-capped green nano-silver), applied onto second degree thermal wounds inflicted onto Swiss Albino mice, was shown to protect the wound from microbial insults while promoted skin regeneration [41]. In the same line, two non-ionic surfactants (e.g., PluroGel^®^ and Wound Dressing and the anti-microbial PluroGel^®^ PSSD) eradicated the pre-formed *P. aeruginosa, Enterococcus* sp., *S. epidermidis, S. aureus* and methicillin-resistant *S. aureus* (MRSA) biofilms on several experimental models, by entrapping and killing the dispersed microorganisms from the biofilm [42]. Similarly, a concentrated gel [loaded with anti-microbial 1% silver sulfadiazine (SSD)] was able to kill pre-formed *S. aureus*, *S. epidermidis*, *MRSA*, *P. aeruginosa* and *E. faecalis* biofilm, eliminate their planktonic cells and prevent intrusion by environmental pathogens, as documented in various in vitro models [43]. In 2016, SSD was tested in a multi-center clinical study, where 70% of the participants experienced positive outcomes (wound closure, reduction of inflammation and odor) with 56% of whom achieved wound closure in less than 11 weeks [44]. Lastly, the sterilization of the consumables used in wound care is also significant. To this end, cotton fibers [coated with nano-structured zinc oxide (ZnO NP) stabilized by the surfactants sodium dodecyl sulfate (SDS) or alkyl hydroxy-ethyl dimethyl ammonium chloride (HY)] were shown to exert strong anti-microbial activity against *E. coli, S. aureus, C. albicans* and *Microsporumcanis*, while they were provento be non-irritable for the skin [45].

Synthetic surfactants and emulsions can also be used as anti-biofilm agents on the surfaces of prosthetic medical devices and health care disposables (e.g., cotton buds and respiratory protective devices). More specifically, nystatin admixed with the surfactant sodium deoxycholate (at a ratio of 1:0.8) induced an over 40% inhibition of *C. albicans* biofilm formation on endotracheal tubes, an effect attributed to the fact that the surfactant enhanced the nystatin penetration into the prosthetics [46]. Regarding the safety of health professionals, engineered respiratory protective devices (with non-woven reusable fabrics and embedded hexamethylene-1,6-bis (N,N-dimethyl-N-dodecyl ammonium bromide; GS-12-6-12 crystals onto halloysite nanocrystals) exerted an anti-microbial action in the presence of water (i.e., humidity from exhaled air) against *E. coli*, *Pseudomonas fluorescens* as well as *Penicillumchrysogenum* and *A. niger* [47]. Lastly, the safety of water in the clinical setting is a very important issue as well. To this end, an anti-microbial and algae-static nanofilm (containing silica-modified quaternary ammonium compounds) was synthesized in an effort to replace chlorine-based water disinfection methods. This nanofilm effectively limited the viability and adhesion of *E. coli* and *Microcystis aeruginosa*, respectively [48].

Emulsions could improve antibiotic efficacy against pathogens without necessarily exerting an antibiotic action, but rather by supporting drug delivery and accumulation. For instance, dipalmitoyl phosphatidyl choline (DPPC) liposomes loaded with the anti-tubercular drugs isioniazid, ethambutol and rifampicin in a ratio of 1:1 (the drug-by-weight ratio was 1:3:2) prolonged the release and alveolar deposition of the antibiotics in mice. Importantly, no hemolytic activity was reported in the human blood samples exposed to the liposomes. Moreover, the mixture remained stable and active after a month-long storage at 4 °C, permitting its use in clinical settings [49]. In this line, ciprofloxacin-loaded polyethylene glycol (PEG)ylated phosphatidylcholine (PC)-rich nanovesicles (phosphatiosomes) coated with soyaethyl morphonium ethosulfate (SME), facilitated the antibiotic release and accumulation of ciprofloxacin in the lungs of a rat model of MRSA lung infection, thus resulting in lower lung bacterial counts compared to the animals that were treated with free ciprofloxacin [50]. In another recent study, a new nano-emulsion (comprised of oils, surfactants and co-surfactants that increased the solubilization of the broad-spectrum antifungal factor sulconazole (SCZ) was synthesized and when tested in SD male rats, showing a higher skin penetration capacity and stronger inhibitory activity against *C. albicans* and *Trichophyton rubrum* compared to the commercial miconazole cream [51]. Likewise, such approaches have been tested for the ocular delivery of anti-microbials as well. A nano-emulsion made from Tween-80, Soluphor^®^ P, ethyl-olate and deionized water was used as a vehicle for the ocular delivery of the antibiotic moxifloxacin, in the irritated eyes of Albino rats. Although the nano-emulsion did not affect the anti-microbial activity of moxifloxacin, it did prolong the contact time and release of the antibiotic in the eye, accelerating the recovery process [52]. In the same manner, nanoparticles carrying ofloxacin (with PEGylated lipids and chitosan) enhanced the drug-mucoadhesion strength that could be linked to better therapeutic outcomes [53]. Table 1 summarizes the anti-microbial effects of surfactants against common pathogens: 

## 4. Anti-Oxidant Properties

Reactive oxygen species (ROS) are the metabolic by-products of both endogenous and exogenous stimuli [54,55] in addition to being important factors of cellular homeostasis through the regulation of signal transduction pathways [56,57]. Under physiological conditions, ROS production and antioxidant defense systems are in balance, but when such production is overwhelming, then it leads to the excessive accumulation of ROS (i.e., oxidative stress) [58,59]. During oxidative stress, ROS can interact with DNA, proteins and lipids, thus contributing to the pathophysiology of various disease mechanisms involved in cancer, aging, diabetes as well as cardiovascular and neurodegenerative disorders [60,61,62]. 

Food products are also susceptible to oxidation, mainly affecting their lipid, protein and carbohydrate content. Specifically, lipid oxidation in emulsion-based food products is considered as the main mechanism causing the deterioration of food quality (e.g., reduced nutrition value), short shelflife, undesirable rancidity, color, texture, etc.). Moreover, the subsequent formation of toxic by-products is often associated with the incidence of pathological conditions including neurodegenerative diseases and cancer [63,64]. To this end, the amelioration of oxidatively-induced damage through the utilization of synthetic and/or naturally-occurring antioxidant compounds, both in humans and in food products, has been extensively reported. In most cases, synthetic antioxidants appear to be more efficient at lower concentrations, while naturally-derived ones usually require higher concentrations. In this context, the utilization of SAAs as novel-antioxidant compounds has attracted scientific interest in recent years [65,66,67]. In general, lipid oxidation in emulsion-based products including those of the food, cosmetic and pharmaceutical industries, is a major health concern for consumers [68]. Specifically, the lipid oxidation of emulsion-based foods, rich in fatty/oils, can deteriorate the food’s nutritional and sensory quality (during processing and storage) in terms of undesirable color, texture and rancidity while the formation of toxic by-products could potentially relate to serious health problems [69]. In general, oil-in-water (O/W) emulsions are used as an emulsion-based model, in food products, consisting of emulsifier-surrounding lipid droplets dispersed in an aqueous continuous phase [70]. Lipid oxidation takes place at the interface between the aqueous and oil phase and it is the net effect of the activity of various enzymes, transition metals and photo-sensitizers, all of which can promote lipid oxidation [71]. Therefore, it is evident that the composition and characteristics of the interfacial layer and the surrounding microenvironment are critical factors, influencing lipid oxidation rates and oxidation stability in O/W systems [72,73,74]. Consequently, the use of interfacial agents able to manipulate interfacial characteristics, has attracted significant attention, especially towards their application in commercial food products. Indeed, several synthetic compounds that possess both surface active and antioxidant potential have been evaluated for their use as antioxidant emulsifiers in O/W emulsions [75]. However, different molecular aspects including the charge [76], length of polar head [77] and the length of the hydrocarbon tail [78] reflect the extent of their antioxidant capacity. 

The visible light irradiation of photosensitizers such as riboflavin (hydrophilic) [74] and chlorophyll (lipophilic) [79], appear to represent a key mechanism of promoting lipid oxidation in food products [80,81]. In a recent study, anionic sodium dodecyl sulfate (SDS) and neutral Tween-20 coated oil droplets, were shown to be more stable compared to those coated with cetyl trimethyl ammonium bromide (CTAB) in riboflavin-photosensitized O/W emulsions [71], although contradictory results were reported in another study [82]. Moreover, lipid oxidation appeared less rapidly in droplets coated with the anionic surfactant SDS, compared to those coated with the non-ionic surfactant Tween-80 [83]. Finally, a study that evaluated the influence of the structure’s interface on the oxidative stability of the surfactant-stabilized O/W emulsions indicated that the metal-initiated lipid oxidation and the formation of volatile compounds occurred more rapidly in the emulsions stabilized by a mixture of non-ionic Tween-20 and Span-20 surfactants, when compared to those stabilized by a single Tween-20 [84].

Non-ionic surfactants (e.g., polysorbates; Tween group) have been widely used in preclinical and clinical practice due to their amphiphilic nature as emulsifiers. Specifically, Tween-20/80 non-ionic surfactants have been used as pharmaceutical surfactants in various drug delivery systems (oral/parenteral) with poor solubility and consequently, their intravenous use has been approved by the European Medicines Agency and US Food and Drug Administration [85]. However, although Tween-20/80 surfactants are generally characterized by a safe profile, extensive knowledge of their antioxidant potential is rather limited. In a recent report, the antioxidant capacity of Tween-20/80 was evaluated by several in vitro assays at concentrations commonly used in laboratory tests [86]. According to the results, both surfactants possessed a low scavenging capacity against (2,2-diphenyl-1-picryl-hydrazyl-hydrate; DPPH) radical formation. However, Tween-20 and -80 significantly inhibited the generation of ROS (by means of phorbol 12-myristate 13-acetate (PMA) or the H_2_O_2_-induced activation of human neutrophils), while Tween-20 exhibited the strongest reductive activity. Specifically, Tween-20 showed a greater inhibitory effect against ROS formation in PMA-induced human leucocytes compared to the H_2_O_2_-treatedcells, while the antioxidant capacity of Tween-80 was similar in both cases. According to the authors of this study, the results indicate that the anti-oxidant potential of both polysorbates (i.e., in terms of decreased ROS production) cannot be assigned to their free radical scavenging ability, but rather to their undefined biological interaction-based mechanism(s) [86]. 

Another type of non-ionic synthetic surfactant [formed by the esterification of Vitamin E in a reaction catalyzed by polyethylene glycol (PEG) 100], is D-α-Tocopheryl polyethylene glycol 1000 succinate (TPGS/Vitamin E TPGS), a water-soluble derivative of natural Vitamin E [87,88]. Being readily miscible with oils as well as an important solubilizer of water-soluble and -insoluble agents, TPGS surfactants have been extensively used in drug delivery systems (DDSs) [89]. Specifically, it has been reported that TPGS surfactants possess a variety of functions as (i) the enhancers of drug solubility [90], (ii) the excipients for multidrug resistance (MDR) and drug absorption [91], (iii) the enhancers of drug cytotoxicity [92] and (iv) anti-tumor agents synergistically acting with other anticancer drugs [93]. Furthermore, these molecules were able to prevent the cyclosporin A (CsA, an immunosuppressive drug)-induced production of ROS and inhibit the loss of protein-bound sulfhydryl groups [94]. 

On the other hand, tyrosol, a biophenol with notable anti-oxidant potential [95], was utilized for the formation of alkyl-succinylatedtyrosol synthetic amphiphilic lipids (catalyzed by lipase succinylation of tyrosol with different alkyl chain lengths of alkyl succinic anhydrides) [96]. According to the characterization profile of tyrosol-based-derived synthetic lipids, all four derivatives exhibited surface active and anti-oxidant abilities to a variable extent. Specifically, among them, the 2-dodecen-1-yl succinylated compound exhibited greater activity against DPPH radical formation when compared to free tyrosol, as well as to the rest of the synthetic lipids. At an emulsion state, both 2-octen-1-ylsuccinylated and 2-dodecen-1-ylsuccinylatedtyrosol-based emulsions inhibited lipid oxidation to a greater extent, as opposed to that of free tyrosol emulsions. 

In another report, erythorbyl laurate (6-O-lauroyl-erythorbic acid), a synthetic product derived from the enzymatic esterification between erythorbic acid (a stereoisomer of L-ascorbic acid widely used as an anti-oxidant in a variety of processed foods) and lauric acid (a medium-chain fatty acid with a strong anti-microbial activity against food-related pathogens) was documented to act as a surface active agent with significant foaming ability [97,98]. Such property was related to its high concentration at the O/W interface and consequently resulted in the significant inhibition of lipid peroxides, particularly in soybean oil emulsions. The observed emulsifying and anti-oxidant properties indicate its potential use as a promising solubilizer/stabilizer of hydrophobic functional food ingredients in emulsified food systems [99].

Chitosan (CS), a semi-synthetic derivative of the polysaccharide chitin (produced through alkaline deacetylation), has exhibited considerable anti-microbial, wound-healing and anti-oxidant activities, among others [100]. However, the low degree of water-solubility is a major disadvantage regarding the efficacy of the compound [101]. To this end, synthetic single N-quaternized (QCS) and double N-diquaternized (DQCS) chitosan derivatives were shown to exhibit water solubility. Moreover, both QCS and DQCS derivatives exhibited higher anti-oxidant capacity when compared to single CS. Specifically, the anti-oxidant potential of all the tested compounds followed the order of DQCS > QCS > CS, as evidenced by various anti-oxidant assays [102]. These results suggest that the DQCS synthetic derivatives of chitosan could be used in pharmaceutical and food areas as promising anti-oxidant agents. On another note, the synthesized derivatives of glucosyl- and glucuronosyl alkyl gallates demonstrated better surfactant and anti-oxidant activities when compared to those of alkyl galates. However, their ROSscavenging capacity appeared to be lower when compared to that of alkyl esters [103]. 

Native as well as the synthetic derivatives of carrageenans have all been shown to associate with significant anti-oxidant potential, such as in the case of κ-carrageenan oligosaccharides as well as some of their over-sulfated (SD), low-, high-acetylated (LAD and HAD, respectively) and phosphorylated (PD) derivatives. According to the results, all the types were shown to limit ROS production, to a variable extent, thereby suggesting that chemical modification(s) could enhance their anti-oxidant potential, in vitro, in a manner following the pattern of PD > SD > LAD > HAD. This observation, in turn, highlights the significant role of the sulfated content in exerting an anti-oxidant response [104]. 

Moreover, a newly synthesized collagen peptide (CP)-κ-carrageenan oligosaccharide complex κ-ca3000 + CP, was evaluated for its potential free radical scavenging activity. To this end, the complex [consisting of κ-carrageenan conjugated with bioactive tilapia skin collagen peptides (CP), rich in hydroxyl and carboxyl groups, and with noted anti-oxidant and photo-protective properties] was effective against UV-induced cell death in HaCaT and MEF cells more efficiently than each compound alone. Finally, this effect was mediated by: (i) the inhibition of apoptotic cell death (HaCaT), (ii) the induced production of collagen I and reduced matrix metalloproteinase-1 (MMP-1) expression levels (MEF), accompanied by (iii) the inhibition of mitogen-activated protein kinases (MAPKs) pathway activation and ROS production as well. According to the authors, the complex κ-ca3000/CP exerts significant photo-protective and anti-oxidant activities, thereby indicating its potential use in cosmetic and pharmaceutical industries [105]. 

Rhamnolipid glycolipids have been shown to have significant antioxidant properties, with a mixed mono and di-rhamnolipid from *Marinobacter litoralis* MB15 showing about a 10% lower DPPH scavenging ability (72.6%) at 5mg/mL than ascorbic acid at the same concentration [106]. Similarly, rhamnolipids were also reported as having significant antioxidant ability by Abdollahi et al., although this was lower than for the artificial food antioxidant butylated hydroxyanisole (BHA) [107]. In a more direct demonstration of the potential of rhamnolipids as antioxidants, Liu et al. [108] compared the ability of rhamnolipids, saponins (a plant derived biosurfactant) and Tween 80 to protect ω-3-fatty acids in polyunsaturated marine oil emulsions. They found that the order of decreasing oxidative stability was rhamnolipids > saponins > Tween 80, highlighting the potential of the biosurfactants. The antioxidant properties of sophorolipids are reported to be weak [109], which may explain the lack of reports on their antioxidant ability. Takahashi et al. 2012 [110] report that all common MEL derivatives (e.g.,MEL A, MEL B and MEL C) have a lower antioxidant ability in DPPH in vitro tests than arbutin, a common positive control known to have strong radical scavenging properties. However, when tested for protection against H_2_O_2_-induced oxidative stress on human skin fibroblasts (NB1RGB cell lines), MEL C surprisingly had a greater protective effect than arbutin. The different responses of glycolipids to different antioxidant tests may also partly explain the dearth of reported antioxidant properties.

Table 2 summarizes the known polymers with documented anti-oxidant activity and the suggested mechanism(s) of action:

## 5. Anti-Viral Properties

Re-emerging and emerging viral infections lacking effective therapeutic and preventive measures constitute a global threat. Vulnerable populations (immune-deficient and/or immunocompromised individuals and groups at the extremes of age) are more prone to developing severe disease manifestations and rare complications, often leading to chronic disorders and death. Currently available anti-virals comprise of: (i) directly-acting (DAAs) and (ii) host-acting (HAAs) anti-virals, which can either directly target the virion or alter thegene expression and enhance host anti-viral responses, respectively [111]. SAAs are interesting candidates for anti-viral DAA or HAA drug discovery, due to their chemical and physical properties. 

One of the first reported applications of synthetic surface-active compounds with anti-viral potential, was their incorporation in topically-applied gels for the management of sexually-transmitted infections. Nonoxynol-9 was the first non-ionic surfactant with established spermicidal activity, which was tested as a potential virucidal against enveloped viruses, including those of human immunodeficiency virus (HIV). Clinical trials that investigated the use of nonoxynol-9 against HIV infection in female sex workers led to controversial results regarding the efficacy and safety of a such approach [112,113]. Although nonoxynol-9 appeared as a non-appropriate candidate for a topical virucidal use, it did set the stage for the research of synthetic SAAs with virucidal potential [114,115]. Of note, the poly-anionic SDS was found to be effective against HIV-1, HSV-2 and the non-enveloped human papilloma virus (HPV), due to its surface-active and denaturing properties [116]. In an earlier study, sodium lauryl sulfate (SLS; an anionic surfactant with protein denaturing potency, otherwise called SDS) was shown to be effective against the same targets. Furthermore, a gel formulation containing SLS was potent against herpes simplex virus type-2 (HSV-2)-induced vaginal and epidermal lesions in rabbits and mice [117]. Anti-herpetic activity was also exhibited by chlorhexidine, a very potent surface-active agent, that acted by destabilizing the viral lipid outer layer [118]. On another note, soaps and sanitizers could also be as readily available for topical virucidal action after viral exposure. For instance, commercial South African soap bars diluted in water demonstrated virucidal action against cell-free HIV-1 virions and cytotoxic effects against HIV-1-infected lymphocytes. These effects were reported after a very short incubation period, ranging from 30 secs to 2 min [119]. Accordingly, a microemulsion formulated from different fractions of Tween-80, Span-20, ethanol, oil, isopropyl myristate (IPM), and distilled water, dramatically limited the infectivity of HSV-2 in vitro [120]. 

The research on the virucidal properties of SAAs, also targets non-sexually transmitted viruses that are related to significant mortality rates in vulnerable groups. For instance, the Ebola virus (EBV) is a serious life threat, causing re-emerging epidemics as there is no specific medication available yet. In the early 2000s, following the African epidemic of the late 1990s, the findings of a study proposed that the nano-emulsion ATB could decrease the Ebola virus strain Zaire concentration by over 4.0 logs in a cell culture medium and surfaces in 20 min. Such a significant decrease in the viral population could potentially lead to its complete eradication [121]. Another serious seasonal epidemic is influenza infections. The efficacy of current anti-viral agents against influenza is doubted, underlining the urgent need for more effective preventive and treatment strategies [122]. Surface sanitization is a crucial factor in limiting influenza spread. Household cleaning disinfectants, branded anti-viral wipes and surfactant-coated silica nanoparticles (SNPs) reportedly limited influenza virus strain H1N1 viability [123]. In particular, SNPs coated with didodecyl dimethyl ammonium bromide (DDAB; a quaternary ammonium cationic surfactant) were effective against the H1N1 influenza strain, in suspension or when immobilized into surfaces, indicating its potential role as an attractive coating factor for large surfaces and prosthetics [124]. Lastly, two nano-emulsions made from soybean oil, tributyl phosphate and Triton X-100, namely 8N8 and 20N10, exhibited prophylactic properties against influenza infection, in both in vitro and in vivo models.Specifically, CD-1 mice that received these mixtures prior to the viral exposure exhibited significantly elevated survival rates compared to mock-treated animals [125]. The emergence of the severe acute respiratory syndrome-coronavirus-2 (SARS-CoV-2) almost six months ago in Wuhan, China, and the consequent coronavirus disease of 2019 (COVID-19) pandemic, has fast-tracked many studies on preventative and therapeutic means. Despite the global efforts, currently, there are no specific drugs and/or vaccines available. Therefore, the public relies on disinfection and sanitization methods to limit the viral spread. Very recent studies showed that SARS-CoV-2 can remain infectious on inanimate objects from 2 h to 9 days, depending on temperature, humidity and the type of material. Ethanol, 2- and 1- propanolare rigorous disinfectants of surfaces and skin, and limit the virus infectivity as much as 5 logs, in just 30 s [126]. Additionally, chlorine-based solutions, that are mainly used for surface and wastewater treatments, have exhibited concentration-depended anti-SARS-CoV-2 effects. Indeed, 1:50 and 1:100 dilutions have been proved to limit the viral load by 3 logs [127].

Viral infections account for a significant percentage of food-borne diseases worldwide. The two most prevalent viral enteropathogens are the norovirus (NoV) and rotavirus (RV). Human NoV (HuNoV) does not propagate in cell cultures, but instead, close phylogenetically viral surrogates, like the murine NoV (MNV), are utilized in research for anti-noroviral compounds. In a recent study, the anti-MNV potential of the surfactants SDS (powder), NP-40 (4-nonylphenyl-polyethylene glycol), Triton X-100, and Tween-20 was examined, and found that all surfactants were able to inactivate MNV-1 in a dose-dependent manner [128]. Similarly, a mixture consisting of levulinic acid and SDS limited the activity of MNV and hepatitis A virus (HAV) on strawberries. The mean reduction of viral yield only reached 1-log, but levulinic acid/SDS mixture was more efficient compared to water or bleach [129]. A recent study, using viral-like particles, consisting of the noroviral capsid proteins only, reported that the cationic surfactant cetyl trimethyl ammonium bromide (CTAB) and the anionic surfactant SDS could disassociate and entrap the viral-like particles in micelles, effectively limiting their infectivity [130]. Contrasting results have been reported concerning the most potent hand disinfection method against MNV. Some have proposed that ethanol-based sanitizers are the most efficient anti-MNV sanitization method [131], while others have shown that common soaps diluted in water are far superior [132,133]. Regardless, alcohol-based sanitizers have been shown to completely diminish the infectivity of RV and HAV in less than 30s [134]. 

Rhamnolipids have been reported to have antiviral activity against the herpes simplex virus (HSV) types I and II [135]. The effect was dose dependent and occurred at concentrations below the critical micelle concentration of the rhamnolipid. One of the first reports on the antiviral activity of SLs was presented by Shah et al. [136]. The authors discovered an anti-human immunodeficiency virus (HIV) activity of the tested SLs together with a spermicidal activity comparable with nonoxynol-9. The potential activity of SLs against retrovirus, herpes virus and papillomavirus has been the subject of several patents [137,138]. Moreover, the report by Gross et al. [137] also presented the influence of SLs on the Epstein–Barr virus. 

Table 3 summarizes the known SAAs with the documented anti-viral activity as well as their potential targets and the suggested mechanism(s) of action:

## 6. Anti-Inflammatory Properties 

Balanced inflammatory responses are crucial for survival. They are divided into two broad categories: acute and chronic. Acute inflammatory responses can be triggered by pathogens, chemical irritants or tissue injury and can result in the neutralization of the stimuli and wound tissue repair [139]. The main soluble mediators of inflammatory response are interleukins (ILs), chemokines, eicosanoids and histamine, produced by the injured tissue or in situ immunological populations. On the other hand, the duration and magnitude of the inflammatory response is tightly regulated by transforming growth factor-β (TGF-β), which locally suppresses the production of pro-inflammatory molecules, whilst supporting the proliferation of fibroblasts and the production of extracellular matrix proteins, involved in the remodeling of the damaged tissue. In this way, unregulated responses can lead to systemic and chronic inflammation that are strictly correlated with autoimmune disorders, cancer, metabolic and cardiovascular disease, among other pathological conditions [139].

Currently, synthetic surfactants are studied as potential drug candidates for the acute respiratory distress syndrome (ARDS). This is a multifactorial condition, also caused by pulmonary surfactant deficit and prolonged inflammatory responses. Importantly, COVID-19 can lead to ARDS as SARS-CoV-2 can infect and impair the function of type II cells in the lungs, affecting the production of pulmonary surfactants, at the third stage of the infection [140]. Current therapeutic approaches include anti-inflammatory agents, vasodilators and surfactant replacement therapy, among others [141,142]. The most commonly used are porcine lung surfactants. However, major limitations in their use include: (i) the difficulty of isolation and (ii) their limited production, thereby reflecting the inability of high-dose administration. To this end, synthetic SAAs with anti-inflammatory potential are being studied for their use as pulmonary surfactant substitutes in ARDS patients. For instance, CHF5633 is a novel synthetic surfactant, consisting of: (i) analogues of surfactant proteins B (SP-B) and C (SP-C) (both possessing changes in size and amino-acid sequence) and (ii) a 1:1 phospholipid mixture of dipalmitoyl phosphatidyl choline (DPPC) and palmitoyl oleyl phoshatidyl glycerol (POPG). SP-B and SP-C are the lipid-associated protein components of the pulmonary surfactant that protect the alveoli shape during breathing. CHF5633 has been thoroughly examined for its ability to elicit an anti-inflammatory response in naive or activated neonatal or adult monocytes. In each model used, CHF5633 decreased the expression of the inflammatory mediators like tumor necrosis factor-α (TNF-α) and interleukin-1β (IL-1β), but had no effect on the expression levels of interleukin-8, -10, Toll-like receptor 2 (TLR2) and 4 (TLR-4) [143,144]. Similarly, the activated CD4+T human lymphocytes that were exposed to CHF5633 exhibited higher transcriptional levels of the anti-inflammatory cytokines, interleukin-4 and -10 [145]. 

On another note, it was previously shown that synthetic dimyristoyl phosphatidyl glycerol (DMPG) exerted its anti-inflammatory potential by silencing TLR-signaling in lipopolysaccharide (LPS)-challenged alveolar macrophages [146]. Moreover, a mixture of DPPC, egg-phosphatidylglycerol (PG), palmitic acid (PA) and SP-CL16(6-28) (a peptide with anti-inflammatory potential) [147] dose-dependently limited interleukin inflammatory mediators in LPS-activated human monocytes [148]. 

In addition, another synthetic surfactant, a mixture of recombinant surfactant protein (rSP)-C, palmitoyl-phosphatidyl-glycerol (PPG) and DPPC also inhibited the release of IL-1β, in U937 human lymphoma cells, by interfering with the ATP-mediated inflammasome activation, in a dose-dependent manner. The authors suggested that the active constituent of the surfactant was DPPC, which acted as an antagonist on non-canonical nicotinic acetylcholine receptors in immune cells [149]. 

Lastly, synthetic galactose-taurine sodium salt has been shown to exhibit surface active properties by halting the pro-inflammatory response in LPS-activated RAW 264.7 macrophages via: (i) the down-regulation of inducible nitric oxide synthase (iNOS) and cyclooxygenase-2 (COX-2) expression levels as well as (ii) the suppression of the activation of nuclear factor-κΒ (NF-κΒ). As a result, the secretion of the inflammatory mediators IL-1β, IL-6 and TNF-α was reduced [150]. 

In vivo studies exploring the anti-inflammatory potential of synthetic SAAs are limited. A protein-free synthetic surfactant prepared from DPPC (i.e., hexadecanol and tyloxapol) exhibited anti-inflammatory properties in rats with LPS-induced lung injury. The surfactant-treated rats showed a lower expression of IL-1β, IL-2, TNF-α, interferon-γ (IFN-γ), monocyte chemoattractant protein-1 (MCP-1) and macrophage inflammatory protein-1β (MIP-1β) (chemokines that recruit monocytes and activate granulocytes, fibroblasts and macrophages in situ, respectively), when compared to the non-treated (control) ones [151]. 

Reports on the anti-inflammatory properties of glycolipid biosurfactants are few, but do suggest potential in applications that exploit these properties. Sophorolipids derived from the yeast of *C. bombicola* reduce the lipopolysaccharide-induced expression of inflammatory cytokines [152]. Moreover, SLs display anti-inflammatory effects in in vitro models of allergic conditions by down regulating important genes in IgE production (BSAP (Pax5), TLR-2, STAT3 and IL-6) [153]. MELs have also been shown to inhibit the secretion of inflammatory mediators (amines such as histamine and serotonin, leukotrienes and cytokines) from mast cells [154]. 

**Figure 2:**
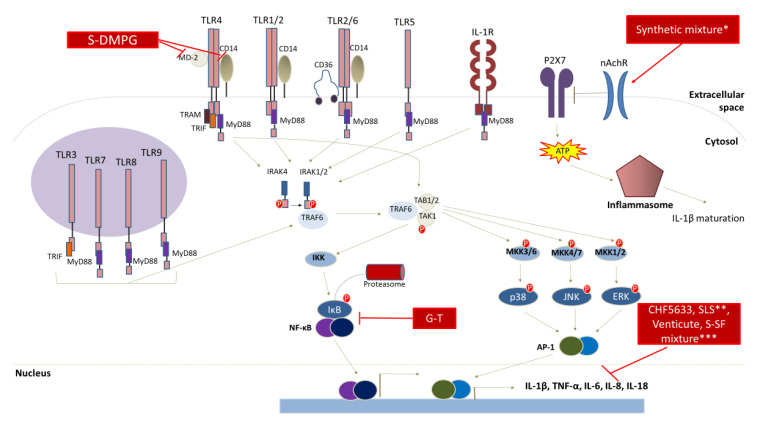
Anti-inflammatory activity of SAAs. Synthetic and biosurfactants exert potent anti-inflammatory effects by manipulating crucial cell signaling events. Specifically, S-DMPG treatments were shown to inhibit TLR-4 signaling, while a synthetic mixture comprised of recombinant surfactant protein (rSP)-C, palmitoyl phosphatidyl glycerol, and dipalmitoyl phosphatidyl choline (DPPC) exerted anti-inflammatory actions by reducing inflammasome signaling. Furthermore, CHF5633, SLS, Venticute and S-SF treatments significantly decreased the production of inflammatory mediators via unknown mechanisms. Synthetic mixture*: synthetic surfactant composed of recombinant surfactant protein (rSP)-C, palmitoyl phosphatidyl glycerol, and dipalmitoyl phosphatidyl choline (DPPC); SLS**: SP-CL16(6-28) and synthetic phospholipid mixture; S-SF mixture***: synthetic surfactant comprised of dipalmitoyl phosphohatidyl choline (DPPC), hexadecanol and tyloxapol. Abbreviations: G-T: synthetic galactose-taurine sodium; S-DMPG: synthetic-dimyristyl phosphatidyl glycerol; P2X7: P2X purinoceptor 7;nAchR: nicotinic acetylcholine receptor; TLR: Toll-like receptor; IL: interleukin; IL-1R: interleukin-1 receptor; MyD88: myeloid differentiation primary response 88; TRIF: toll/interleukin-1 receptor-like protein (TIR)-domain-containing adaptor-inducing interferon-β; ΤRAM: TRIF-related adaptor molecule; IRAK: IL-1 receptor associated kinase; TRAF: tumor necrosis factor receptor-associated factor; TAB1/2: tumor growth factor-β (TGF-β)-activated kinase 1; TAK: TGF-β activated kinase 1; IKK: IκΒ kinase; MKK: mitogen activated protein (MAP) kinase; NF-κΒ: nuclear factor κΒ; ΕRK: extracellular signal activated kinase; JNK: c-Jun N-terminal kinase; AP-1: activator protein-1.

Figure 2 summarizes the molecular targets of surfactants with a documented anti-inflammatory potential: 

## 7. Anti-Cancer Properties

Cancer is considered a multistage disease with multifactorial aetiology and is associated with high global incidence and mortality rates. Chemotherapy, surgery and radiation are still the most common treatment options for malignant tumors. However, all of them are associated with adverse-side effects, thus indicating the lack of specificity and the need for discovering new anti-tumor agents from various environmental resources [155,156,157]. To these ends, only recently, several studies have indicated the anti-tumor potential of various different SAAs.

Benzethonium chloride (BCl) is a synthetic quaternary ammonium salt, characterized as a cationic surfactant and is mainly known for its anti-microbial activities [158]. However, according to the National Cancer Institute/NIH Developmental Therapeutics Program (NCI/NIH-DTP), BCl was also found to exhibit significant anti-cancer potential against leukemia, lung, colon, melanoma and ovarian cancers [159]. In addition, it induced apoptosis in Jurkat T-lymphoma cells, through the release of cytochrome c and the activation of caspases -3 and -9 [158]. In another report, BCl inhibited the proliferative activity of human laryngeal (UMSCC23) and tongue (HN12) squamous carcinoma cells, by inducing a terminal unfolded protein response (UPR), which led to the up-regulation of the transcription factor CCAAT-enhancer-binding protein homologous protein (CHOP) and subsequently to CHOP-dependent apoptosis [160]. This compound also exhibited a significant anticancer effect, against FaDu, (hypopharyngeal) and C666-1 (nasopharyngeal) squamous cancer cells. Specifically, in FaDu cells, BCl-induced apoptotic death was related to the activation of caspases -2, -8, -9, -3 and the loss of mitochondrial membrane potential (Δ*Ψ*m). Moreover, itsin vivo anti-cancer effect was accompanied by the inhibition of FaDu xenografts in SCID (severe combined immunodeficient) mice [159]. 

Finally, another chemical surfactant, the non-ionic NP-40, caused a cytotoxic effect against SK-N-SH human neuroblastoma cells by promoting apoptosis through: (i) the generation of ROS, (ii) the increase in the Bax:Bcl-2 ratio, (iii) the up-regulation of the p53 expression levels and finally (iv) the release of cytochrome c [161]. 

**Figure 3:**
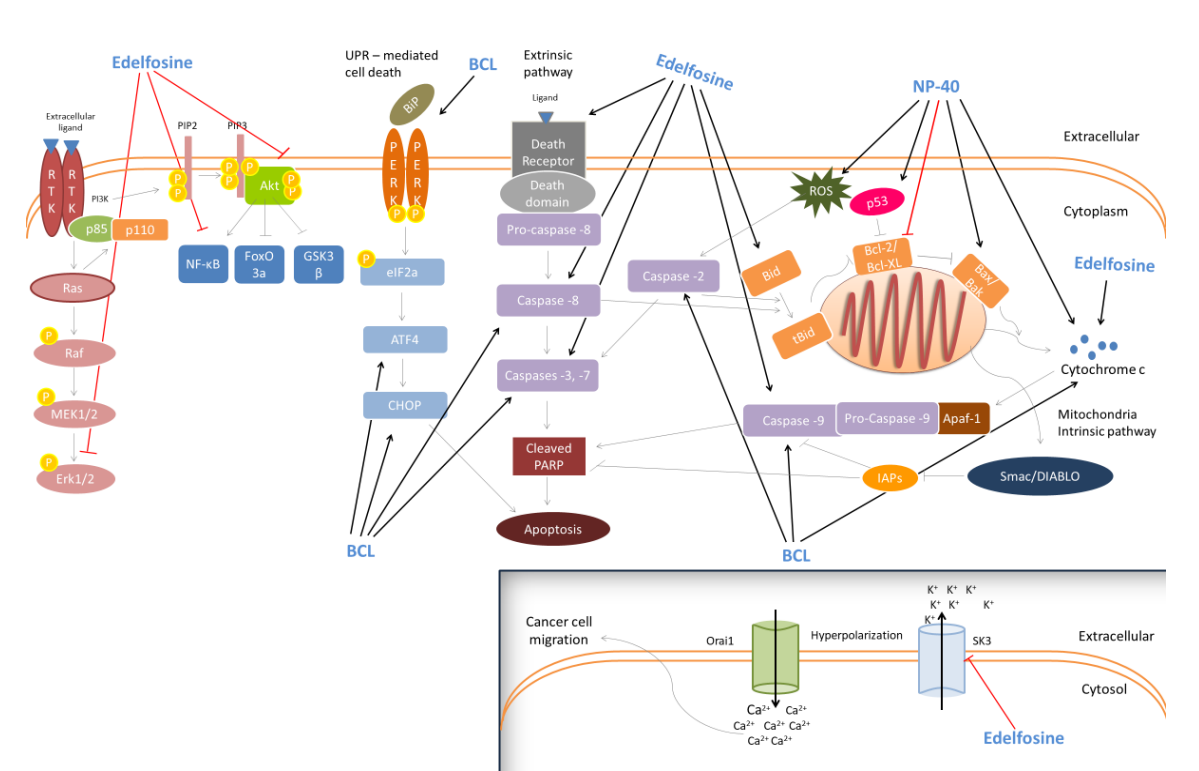
Anti-cancer activity of the synthetic SAs. Synthetic surfactants demonstrate significant anti-cancer activity through the activation of both intrinsic (BCL, Edelfosine, NP-40) and extrinsic apoptotic pathways (BCL, Edelfosine), unfolded protein response-mediated cell death (Edelfosine) and the suppression of the MAPK/ERK and Akt/PI3K signaling pathways (Edelfosine). Additionally, the synthetic SAAs Edelfosine was shown to inhibit the migration of cancer cells by suppressing the small conductance calcium-activated potassium channel 3 (SK3) activity. Abbreviations: RTK: receptor tyrosine kinase; PI3K: phosphatidylinositol-3-Kinase; MAPK: mitogen-activated protein kinase; ERK: extracellular signal-regulated kinase; MEK: MAPK/ERK kinase; PIP_2_: phosphatidylinositol 4,5-bisphosphate; PIP_3_: phosphatidylinositol (3,4,5)-trisphosphate; NF-kB: nuclear factor kappa-light-chain-enhancer of activated B cells; FOXO3a: forkhead box O3a; GSK3β: glycogen synthase kinase 3 beta; UPR-mediated cell death: unfolded protein response-mediated cell death; BiP: binding immunoglobulin protein; PERK:protein kinase R-like endoplasmic reticulum kinase; eIF2a: eukaryotic translation initiation factor 2a; ATF4: activating transcription factor 4; CHOP: CCAAT-enhancer-binding protein homologous protein; PARP: poly (ADP-ribose) polymerase; Bid: BH3-interacting domain death agonist; tBid: truncated Bid; Bcl-2: B-cell lymphoma 2; BCL-XL: B-cell lymphoma-extra-large; BAX: BCL2 associated X; BAK: BCL2-antagonist/killer; ROS: reactive oxygen species; Smac/DIABLO: second mitochondria-derived activator of caspase/direct inhibitor of apoptosis-binding protein with low pI; IAPs: inhibitors of apoptosis proteins; Apaf-1: apoptotic protease activating factor 1; SK3: small conductance calcium-activated potassium channel 3 (SK3); Orai1: ORAI calcium release-activated calcium modulator 1.

Edelfosine (1-*O*-octadecyl-2-*O*-methyl-*rac*-glycero-3-phosphocholine; ET-18-OCH_3_) is a synthetic surface-active alkyl-lysophospholipid which is also considered as a selective anti-tumor agent [162]. In a previous study, edelfosine induced apoptosis through loss of Δ*Ψ*m potential and caspase-3 activation in both Jurkat (T-lymphoid leukemia) and HL-60 (acute leukemia) cells [163]. Moreover, it was shown to induce apoptosis in MM144, MMIS, MM1R (human multiple myeloma) and T-lymphoid Jurkat/B-lymphoid JY (leukemia) cells. Specifically, it promoted apoptosis in MM144 multiple myeloma cells via the recruitment of the Fas/CD95 death receptor and TRAIL receptors DR4, DR5. In this way, the recruitment of Bid (a linker molecule between death receptor and mitochondria apoptotic pathways) into lipid rafts (membrane microdomains, enriched in cholesterol, sphingolipids and gangliosides) facilitated the Fas/CD95- and mitochondria-mediated apoptotic death [164]. A similar anti-cancer mechanism was reported against Jurkat cells [165] and CD138+ malignant cells derived from multiple myeloma patients. [166]. Additionally, edelfosine was found to enhance the sensitivity of SNU638 and AGS gastric cancer cells, against recombinant human tumor necrosis factor (TNF)-related apoptosis-inducing ligand (rhTRAIL)-induced apoptosis. The underlying anti-cancer mechanism appears to involve the up-regulation and/or translocation of death receptor 5 (DR5) into lipid rafts and the kinase p38-mediated loss of Δ*Ψ*m [167]. Moreover, the compound was shown to induce apoptosis in U118 glioblastoma cells through the activation of caspases-3 and -8 and the disruption of ΔΨm, followed by caspase-9 activation [168]. In addition, H-ras-transformed human breast epithelial cells (MCF10-*ras*) were also led to apoptosis, following edelfosine treatment, viathe inhibition of ERK1/2 and Akt kinases activity and the reduction of nuclear factor-kappa B (NF-κΒ) expression levels, probably through the induction of a cyclooxygenase-2 (COX-2) expression [169]. Likewise, edelfosine was able to induce apoptotic death in another two prostate cancer cell lines, LNCaP and VCap, through the differential regulation of p-AKT and activated transcription factor 3 (ATF3), respectively [two known regulators of androgen receptor (AR) activity]. Moreover, the in vivo anti-tumor activity of edelfosine was confirmed in prostate LNCaP [170] and glioma C6 [171] xenografts as well. Finally, a recent report indicated an in vitro anti-metastatic capacity of edelfosine, against RT112 muscle-invasive urothelial cancer cells, mediated through the inhibition of the small conductance calcium-activated potassium channel 3 (SK3; a metastasis inducer). Similarly, edelfosine inhibited the invasive ability of RT112-derived tumors, in vivo, using a modified chicken chorioallantoic membrane (CAM) model [172]. Figure 3 illustrates suggested the anti-cancer mechanisms activated by the different synthetic surfactants. 

The effect of rhamnolipids on the cancer cell lines HL-60, BV-173, SKW-3, and JMSU-1 has been determined for the C10C10 monorhamnolipid R1, and the corresponding C10C10 dirhamnolipid R2 [173]. R1 was the more potent of the two and inhibited cellular division at lower concentrations (IC_50_) than R2. The mechanism of inhibition appeared to be the induction of apoptosis. Rahimi et al. [174] confirmed the anti-cancer properties of R1 and R2 (on the MCF7 human breast cancer cells) and the lower IC_50_ of R1 (25.87 µg/mL) compared to R2 (31.00 µg/mL). Others have also confirmed the anticancer potential of rhamnolipids [175].

Sophorolipids extracted from the yeast strain *Wickerhamiella domercqiae* Y2A isolated from oil-contaminated wastewater were noticed to present anticancer activity as described by Chen et al. [176]. SL was tested against four cancer cell lines, H7402 (liver), A549 (lung), HL60 and K562 (leukaemia) and showed a significant inhibitory effect on cell proliferation on all the tested cell lines. What is more, the authors observed a direct dependence of inhibition on the surfactant’s concentration, where 0% cell viability was recorded for the sophorolipid concentration above 62.5 µg mL^−1^. A cytotoxic effect of lactonic SLs against breast adenocarcinoma lines MDA-MB-231 was described by Ribeiro et al. [177]. Moreover, a correlation between a cytotoxic effect and surfactant’s critical micelle concentration CMC was observed, in other words, when the surfactant’s concentrations were in the range of its CMC, a higher cytotoxic effect was observed. The authors observed a greater anticancer effect of diacetylated lactonic SLs compared to acidic SLs. Similar conclusions were drawn by Shao et al. [178] who tested the effects of ten structurally different SLs on two human oesophageal cancer cell lines, KYSE 109 and KYSE 450. They observed the diacetylated lactonic SL to be the most efficient at inhibiting two oesophageal cancer cell lines, while acidic sophorolipids hardly indicated any anticancer activity. SLs have also shown anticancer activities towards the human cervical cancer cell line HeLa, as presented by Nawale et al. [179] and Li et al. [180] The latter also presented a relationship between the cytotoxicity of diacetylated lactonic SLs of different unsaturation degrees and their carbon chain length. The longer the chain length, the stronger the cytotoxicity of SLs observed. Apart from the aforementioned anticancer activities towards breast, cervical, lung, liver and oesophageal cancer, SLs were also found to show cytotoxic properties against human pancreatic cancer cell lines [181] and glioma cell line LN-229 [182].

There are numerous reports on the anti-tumour activity of MELs against various cancer types. In some, the successful treatment was related to the MEL-containing liposomes as summarised by Silva Coelho et al. [183]. The anticancer effect of MEL-A and MEL-B on human leukaemia HL-60 cell lines was reported by Isoda et al. [184] The surfactants were also found to inhibit the insulin-dependent proliferation of human myelogenous leukaemia cell line K562 through the inhibition of the tyrosine phosphorylation [185,186]. Zhao et al. [187] reported an induction of apoptosis by MELs against mouse melanoma B16 cell lines. The authors [188] later showed a MEL-induced differentiation of melanoma B16 cells takes place through a pathway involving protein kinase Cα (PKCα). In their latest studies, Bakur et al. [189,190] presented the use of MELs for synthesizing gold nanoparticles (Au-NPs) and assessed the MEL-AuNPs properties. Based on their findings, the newly synthesized nanoparticles showed potential cytotoxic activity against human liver hepatoma HepG2 cells. Furthermore, the authors evaluated other MEL-containing nanocomposites, MEL@ZnONPs and Ag–ZnO/MEL/GA, for their cytotoxic activity against HepG2, and observed a decrease in the viability of cells with increasing concentrations of the nanocomposites.

## 8. Anti-Aging Properties

Aging is generally considered a complex, multifactorial and time-dependent process that is strictly related to morbidity and mortality among humans [191]. Specifically, aging is characterized by a significant decline/deterioration of physiological and anatomical functions at the molecular, cellular and tissue levels [192], leading to the impairment of homeostasis and ultimately to death [193]. At the cellular level, several mechanisms have been associated with the aging process including: (i) increased levels of oxidative stress, (ii) the dysfunction of the mitochondrial machinery, (iii) telomere attrition and finally (iv) the impairment of DNA repair mechanism [194,195,196], all of which have been correlated to the onset of different age-related diseases such as cancer, diabetes, cardiovascular disorders and neurodegenerative diseases [197,198,199].

In general, the skin aging process is caused by both intrinsic (chronological aging) and extrinsic factors. It is a rather complex process that is associated with the gradual loss of skin integrity as well as stability, and the decline of its physiological function [200,201,202,203]. Its clinical manifestations include alterations of pigmentation/elasticity and the appearance of wrinkles and dryness, among others [204,205,206]. Specifically, among various environmental (extrinsic) causing factors, exposure to UV radiation is considered as the most prominent one. In addition, extrinsic skin aging is also referred as to photo-aging which further depends on the frequency, duration and intensity of exposure to sunlight [207]. UV-induced photo-aging is mainly mediated by the up-regulation of several matrix metalloproteinases [MMPs; responsible for the degradation of the extracellular matrix (ECM) components] such as collagen, fibronectin and elastin, that in turn, support the skin architecture at a structural and functional level [208,209]. In addition, the reduced expression levels of the tissue inhibitor of metalloproteinases (TIMPs) as well as the altered activity of UV-induced ROS, MAPKs and transcription factors [e.g., nuclear factor κB (NF-κΒ) and activator protein 1 (AP-1)] appear as major mediators of the photoaging process [210,211,212]. In this context, anti-aging and anti-photoaging skin products dominate the cosmetic and/or cosmeceutical industry, reflecting the high demand for developing new and more effective products with such functionality [213,214]. Interestingly, a variety of scientific reports indicate the ability of SAAs as potential molecules capable of preventing and/or ameliorating the symptoms of aging and/or photoaging processes, in different experimental models.

Different studies have shown the protective activity of synthetic biosurfactants against UV-induced damage by utilizing both in vitro and in vivo experimental models. For instance, chitooligosaccharides (COS; water-soluble derivatives of chitin or chitosan) obtained through chemical or enzymatic hydrolysis [215], exerted a significant protective role against UV-irradiated human dermal fibroblasts (HDF). Specifically, 3-5kDa COS were shown to absorb both UV-A and UV-B light, indicating a protective effect against skin photo-damage. At the molecular level, COS inhibited the expression of various MMPs including MMPs-1, -8 and -13 collagenases and MMPs-2 and -9 gelatinases, an effect accompanied by the induction of their negative regulators TIMP-1 and -2. Moreover, COS-treated HDF cells showed an increased expression of different pro-procollagen members, such as I, III and IV, through the negative regulation of the AP-1 signaling pathway members, like c-Jun and c-Fos [216]. Similarly, COS exhibited significant photo-protective activity against UV-induced damage in hairless mouse dorsal skin. Following the topical application of COS, an improvement of UV-induced damages of the skin (at the macroscopic and histopathological levels) were documented and shown to be mediated by the increased levels of total collagen as well as collagen I. Moreover, the increased levels of various anti-oxidant enzymes like catalase (CAT), superoxide dismutase (SOD), glutathione peroxidase (GSH-Px), together with reduced levels of pro-inflammatory cytokines (e.g., TNF-a, IL-1ß and IL-6), contributed to the photo-protective effect of COS [217]. Such results suggest that COS could be used as potential cosmetic agents against the impairment of collagen synthesis in photo-damaged skin. 

Furthermore, a synthetic complex consisting of κ-carrageenan and collagen peptides (CP) (κ-ca3000 + CP) inhibited UV-induced apoptotic death in HaCaT and MEF cells by (i) blocking the activation of the MAPK signaling pathway, (ii) reducing the MMP-1 expression and (iii) inhibiting ROS production, thus resulting in increased levels of collagen I synthesis [105].

Over the years, the prevention of aging has attracted significant interest among the scientific community. On the other hand, the increased production of ROS has been identified as one of the most prominent determinants of aging, affecting cellular anti-oxidant defense systems and favoring pro-inflammatory and immune dysfunction [218,219]. To this end, the development and use of anti-oxidant and anti-inflammatory agents appear as a promising strategy against aging and age-related diseases. Interestingly, COS exert significant in vivo anti-aging activity against D-gal induced subacute aging mice by (i) blocking the impairment of kidney and liver functions, (ii) partially mediating an improvement of the anti-oxidant activity (by the means of the increased expression of CAT, GSH-Px, SOD) and furthermore (iii) enhancing the immune function (by the means of an increased expression of serum immunoglobulins IgG and/or IgM) [220]. 

Glycolipid biosurfactants have been reported to have potential anti-aging properties that could be exploited in cosmetic formulations. The focus of this research has been on MELs, although any glycolipid displaying anti-oxidant or anti-inflammatory properties has anti-aging potential, with rhamnolipids as the subject of patents for anti-wrinkle and anti-aging products [221]. MELs have moisturizing properties comparable to natural skins ceramides [222,223]. Moreover, the ability of MELs to protect skin cells against oxidative stress [110], a property believed to arise from the unsaturated fatty acid chains in MELs, also strongly suggests potential as an anti-aging ingredient.

Table 4 summarizes the known SAAs with documented anti-aging/photoaging activity including the suggested mechanism(s) of action:

## 9. Conclusions

SAAs are molecules of multifunctional significance given their widespread use in almost every major industrial sector producing consumer products, aiming to optimize their utilization in accordance to their beneficial properties. To this end, SAAs are characterized in the context of their health-promoting properties, while the scientific interest keeps on growing in an attempt to shed light on the underlying molecular mechanism(s) by which they exert such properties. This is of utmost importance as it provides a valid rationale for the selection of better chemical derivatives with enhanced functionality. In this respect, biosurfactants have excellent promise as the replacers of synthetic SAAs as they would find favor with consumers due to their perceived naturalness, lower toxicity and ecotoxicity, higher biodegradability and excellent surface active properties. However, the study of their bioactive properties is relatively new and before they can fully replace synthetic SAAs in bioactive applications, more work is required to fully understand the mechanisms and extent of their efficacy in health-promoting activities.

## Figures and Tables

**Table 1:** Anti-microbial actions of the synthetic and biosurfactant SAAs.

Polymer	Pathogen	MIC	MBIC	References
Hexameric with amide moieties (PAHB)	*E. coli*	0.93 μΜ	NA	[8]
Alkylbenzyldimethylammonium chlorides (BAC) analogues	*S. aureus*	2.6–10.6 μΜ	NA	[12]
	*E. faecalis*	3.5–10.6 μΜ	NA	
	*E. coli*	27.9–123.7 μΜ	NA	
	*P.aeruginosa*	223.1–247.5 μΜ	NA	
	*C. albicans*	13.9–123.7 μΜ	NA	
Clorexhidine	*S. anginosus*	0.97–3.91 μg/mL	15.63–31.25 μg/mL	[30]
	*S. constellatus*	0.97–3.91 μg/mL	7.81–31.25 μg/mL	
	*S. gordonii*	1.95–7.81 μg/mL	7.81–31.25 μg/mL	
	*S. mitis*	1.95–15.63 μg/mL	3.91–31.25 μg/mL	
	*S. mutans*	0.12–0.97 μg/mL	15.63–31.25 μg/mL	
	*S. oralis*	0.97–7.81 μg/mL	3.91–15.63 μg/mL	
	*S. salivarius*	0.48–3.91 μg/mL	1.95–15.63 μg/mL	
	*S. sanguinis*	1.95–3.91 μg/mL	7.81–31.25 μg/mL	
Cetylpyridium chloride	*S. anginosus*	0.12–1.95 μg/mL	3.91–15.63 μg/mL	
	*S. constellatus*	0.06–0.48 μg/mL	3.91–31.25 μg/mL	
	*S. gordonii*	0.12–0.97 μg/mL	3.91–31.25 μg/mL	
	*S. mitis*	0.12–0.97 μg/mL	3.91–31.25 μg/mL	
	*S. mutans*	0.06–0.48 μg/mL	3.91–31.25 μg/mL	
	*S. oralis*	0.12–1.95 μg/mL	3.91–31.25 μg/mL	
	*S. salivarius*	0.06–0.48 μg/mL	3.91–15.63 μg/mL	
	*S. sanguinis*	0.12–0.48 μg/mL	3.91–31.25 μg/mL	
Peptide gemini (PG)surfactants	*E. coli*	8–512 μg/mL	NA	[15]
	*S. typhimurium*	8–512 μg/mL	NA	
	*S. aureus*	4–512μg/mL	NA	
	*B. subtilis*	4–512 μg/mL	NA	
Na-lauroyl-arginine methyle ester-derived cationic double-chain surfactants (LANHC_x_)	*M. luteus* ATCC 9341	17–243 μΜ	NA	[14]
	*B. subtilis* ATCC9341	19–486 μΜ	NA	
	*S. aureus* ATCC29213	17–486 μΜ	NA	
	*S. epiermidis* ATCC12228	17–510 μΜ	NA	
	*P. aeruginosa* ATCC27853	68–78 μΜ	NA	
	*E. coli* ATCC25922	38–269 μΜ	NA	
	*K. pneumonea* ATCC 13883	78 μΜ	NA	
	*C. albicans* ATCC10231	19–243 μΜ	NA	
	*C. albicans*	35–436 μΜ	NA	
	*P. aeruginosa*	218–459 μΜ	NA	
	*K. pneumonieae*	218–459 μΜ	NA	
	*E. coli*	166–331 μΜ	NA	
Gemini alanine-derived ammonium salts (bromides and chlorides)	*C. cerevisiae*	40–1200 μΜ	NA	[34]
	*C.albicans*	80–1200 μΜ	NA	
	*Rhodotorulamucilaginosa*	10–1200 μΜ	NA	
Rhamnolipid (RL)	*P. aeruginosa PAO1*	NA	NA	[16]
	*E. coli NCTC10418*	NA	NA	
	*B. subtilis NCTC10400*	0.5% (*w*/*v*)	NA	
	*S. aureus ATCC9144*	0.5% (*w*/*v*)	NA	
	*E. coli*	12–16 mg/mL	NA	[17]
	*P. aeruginosa*	8–12 mg/mL	NA	
	*S. aureus*	16–24 mg/mL	NA	
	*B. cereus*	12–16 mg/mL	NA	
	*K. pneomoniae*	16–24 mg/mL	NA	
	*C. albicans*	8–12 mg/mL	NA	
	*C. krusei*	12–16 mg/mL	NA	
	*L. monocytogenes*	78.1– >2500 µg/mL	NA	[19]
	*B. cereus*	pH 5 no growth pH 6 no growth pH 7 19.5 µg/mL pH 8 78.1 µg/mL pH 9 156.2 µg/mL	NA	[18]
	*L. moncytogenes*	pH 5 no growth pH 6 19.5 µg/mL pH 7 156.2 µg/mL pH 8 > 2500 µg/mL pH 9 >2500 µg/mL	NA	
	*S. aureus*	pH 5 9.8 µg/mL pH 6 39.1 µg/mL pH 7 > 2500 µg/mL pH 8 > 2500 µg/mL pH 9 > 2500 µg/mL	NA	
	*E. coli*	pH 5 > 2500 µ g/mL pH 6 > 2500 µg/mL pH 7 > 2500 µg/mL pH 8 > 2500 µg/mL pH 9 > 2500 µg/mL	NA	
	*S. enterica*	pH 5 > 2500 µg/mL pH 6 > 2500 µg/mL pH 7 > 2500 µg/mL pH 8 > 2500 µg/mL pH 9 > 2500 µg/mL	NA	
	*S. mutans*	Mixed RL 390 mg/mL MonoRL 780 mg/mL diRL 390 mg/mL	NA	[20]
	*S. oralis*	Mixed RL 97.5 mg/mL monoRL 780 mg/mL diRL 780 mg/mL	NA	
	*S. sanguinis*	Mixed RL 97.5 mg/mL monoRL 780 mg/mL diRL 390 mg/mL	NA	
	*A. naeslundii*	Mixed RL 195 mg/mL monoRL 1560 mg/mL diRL 780 mg/mL	NA	
	*N. mucosa*	Mixed RL 390 mg/mL monoRL 780 mg/mL diRL 390 mg/mL	NA	
MELs	*B. Cereus*	1.25 mg/mL	NA	[26]

MIC: minimum inhibitory concentration; MBIC: minimum biofilm inhibitory concentration; NA: non-applicable.

**Table 2:** Anti-oxidant activity of SAAs.

SAAs	Assay	Mechanism of Action	References
SDS (anionic) Tween-20 (non-ionic) CTAB (cationic) surfactants, O/W emulsions	Lipid hydroperoxides	↓ lipid oxidation	[71]
SDS (anionic) Tween-80 (non-ionic) surfactants, O/W emulsions	Propanal/hydroperoxides	↓ lipid oxidation	[83]
Tween-20 (non-ionic) Tween-20 (non-ionic/Span-20 (co-surfactant) mixture, O/W emulsions	oxygen uptake, conjugated dienes (CD) and formation of volatile compounds	↓ lipid oxidation	[84]
Tween-20/Tween-80 (non-ionic) surfactants	DCFH-DA / Flow Cytometry	↓ ROS productionin PMA/H_2_O_2_-treated human neutrophils	[86]
D-α-Tocopheryl polyethylene glycol 1000 succinate (TPGS/Vitamin E TPGS)	TBARS	↓ ROS production	[94]
Alkyl succinylatedtyrosol synthetic amphiphilic lipids	DPPH radical scavenging, TBARS	↓ formation of DPPH radicals ↓ lipid oxidation	[96]
Erythorbyl laurate (6-O-lauroyl-erythorbic acid),	TBARS	↓ lipid oxidation	[99]
single N-quaternized (QCS) and double N-diquaternized (DQCS) chitosan	DPPH, hydroxyl, superoxide radicals, reducing power	↓ formation of DPPH, hydroxyl and superoxide radicals ↑ reducing power	[102]
Glucosyl- and glucuronosyl alkyl gallates derivatives	DPPH, FRAP	↓ formation of DPPH, ↑ rates of reduction of Fe^3+^ to Fe^2+^	[103]
κ-carrageenan oligosaccharides, oversulfated (SD) low acetylated (LAD) high acetylated (HAD) phosphorylated (PD) κ-carrageenans	DPPH, superoxide anion, hydroxyl radical scavenging activity	↓ formation of DPPH, superoxide and hydroxyl radicals	[104]
κ-ca3000 + CP complex (κ-carrageenan 3kD+ collagen peptides)	DCFH-DA/FlowCytometry	↓ ROS production Protection of HaCaT abd MEF cells from UV-induced cell death	[105]
Rhamnolipids	DPPH	↓ formation of DPPH	[106]
	DPPH, FRAP	↓ formation of DPPH, ↑ rates of reduction of Fe3+ to Fe2+	[107]
	TBARS, Lipid hydroperoxides	↓ lipid oxidation	[108]
Sophorolipids	DPPH, FRAP	↓ formation of DPPH, ↑ rates of reduction of Fe3+ to Fe2+	[109]
MELs	DPPH, superoxide anion scavenging activity	↓ formation of DPPHand superoxide radicals	[110]

SDS: sodium dodecyl sulfate; CTAB: cetyl trimethyl ammonium bromide; O/W: oil in water; ROS: reactive oxygen species; PMA: phorbol myristate acetate; CP: collagen peptide; MELs: mannosylerythritol lipids; DCFH-DA: dichloro-dihydro-fluorescein diacetate; TBARS: thiobarbituric acid reactive substances; FRAP: ferric reducing antioxidant power; DPPH: 2,2-dipheny;-1-picrylhydrazyl; ↑: increased; ↓ decreased.

**Table 3:** Anti-viral activity of SAAs.

SAA	Mechanism of Action	Viral Target	Experimental Model	References
SDS/SLS (anionic surfactant)	Membrane disruption and capsid denaturation	HIV 1 and 2, HPV	various cell lines	[116]
	Membrane disruption and capsid denaturation	HIV 1 and 2, HPV	various cell lines	[117]
		HSV 2	in vivo (hairless mice)	
SDS (anionic surfactant),levulinic acid and sodium hypochlorite	Undefined	MNV, HAV	in vitro	[129]
CTAB (cationic surfactant), SDS (anionic surfactant)	Particle disassociation and entrapment in micelles	MNV	in vivo	[130]
Tween-20 (non-ionic surfactant), Triton X-100 (non-ionic surfactant) NP-40 (non-ionic surfactant)	Capsid denaturation	MNV	RAW 264.7 murine macrophages	[128]
Chlorhexidine (cationic surfactant)	Membrane disruption	Herpes virus	hep-2 and RK_13_ cells	[118]
African soap bars diluted in water	Undefined	Cell-free HIV-1, HIV-1 infected lymphocytes	human PBMCs	[119]
Common soaps	Undefined	MNV-1, HuNoV GI.4 and GII.4	in vivo	[134]
Micro-emulsion of Tween-80 (non-ionic surfactant and emulsifier), Span-20 (co-surfactant), ethanol, oil, isopropyl myristate (IPM), and distilled water	Undefined	HSV-2	vero cells	[120]
Nano-emulsion ATB	Undefined	Cell culture medium and surfaces	vero cells	[121]
Nano-emulsion of soybean oil, tributyl phosphate AND Triton X-100 (non-ionic surfactant)	Limited infectivity in animals	Murine influenza A virus	in vivo (CD-1 mice)	[125]
Nanoparticles coated with DDAB (cationic surfactant)	Structural damage and inactivation	H1N1 influenza strain	MDCK cells	[124]
Rhamnolipid	Not defined	HSV 1 and 2	MDBK cells	[135]
Sophorolipid	Membrane disruption	HIV	cell-free HIV inactivation model	[136]

SLS: sodium lauryl sulfate; HIV: human immunodeficiency virus; HPV: human papilloma virus; HSV: herpes simplex virus; MNV: murine norovirus; HAV: hepatitis A virus; RK: rabbit kidney; HuNoV: human norovirus; DDAB: didodecyl dimethyl ammonium bromide; MDBK: Madin-Darby bovine kidney; PBMCs: peripheral blood mononuclear cells; MDCK: Madin–Darby canine kidney.

**Table 4:** Anti-aging/photoaging activity of SAAs.

SAA	Experimental Model	Mechanism(s) of Action	References
Chito-oligosaccharides 3-5kDa (COS)	UV-A/B-irradiated human dermal fibroblasts (HDF)()UV-A/B-irradiated hairless BALB/c mice D-gal induced subacute aging mice (Kunming mice)	↓ MMP-1/8/13 ↓ MMP-2/9 ↓AP-1, c-Jun, c-Fos ↑ TIMP-1/2 ↑ procollagen I-III-IV Improvement of macroscopic and histopathological features ↑ total collagen and collagen I ↑CAT, SOD, GSH-Px↓TNF-a, IL-1β, IL-6 Improvement of kidney and liver function ↓ALT, AST, ALP, UA, CREA ↑ CAT, GSH-Px, SOD ↑IgG/IgM	[215,216,217]
κ-ca3000 + CP	UV-A/B-irradiated HaCaT and MEF cells	↓ UV-induced apoptotic death Inhibition of MAPK pathway and ROS production ↓ MMP-1	[105]

ALT: alanine transaminase; AST: aspartate transaminase; ALP: alkaline phosphatase; UA: uric acid; CREA: creatinine; ↑ increased; ↓ decreased.
